# Ileostomy or ileal pouch-anal anastomosis for ulcerative colitis: patient participation and decisional needs

**DOI:** 10.1186/s12876-021-01916-0

**Published:** 2021-09-19

**Authors:** Jessica N. Cohan, Elissa M. Ozanne, Rebecca K. Hofer, Yvonne M. Kelly, Anna Kata, Craig Larsen, Emily Finlayson

**Affiliations:** 1 Department of Surgery, University of Utah, 30 North 1900 East, Salt Lake City, UT 84132 USA; 2 Department of Population Health Sciences, University of Utah, Salt Lake City, UT USA; 3 Department of Family and Community Medicine, University of California, San Francisco, CA USA; 4 Department of Surgery, University of California, San Francisco, CA USA; 5 Department of Surgery, Medstar Georgetown University Hospital, Washington, DC USA; 6 Department of Surgery, New York Presbyterian-Queens, Flushing, NY USA

**Keywords:** Ulcerative colitis, Shared decision making, Ileal pouch-anal anastomosis, Ileostomy, Qualitative research

## Abstract

**Background:**

Up to 30% of patients with ulcerative colitis will undergo surgery resulting in an ileal pouch-anal anastomosis (IPAA) or permanent end ileostomy (EI). We aimed to understand how patients decide between these two options.

**Methods:**

We performed semi-structured interviews with ulcerative colitis patients who underwent surgery. Areas of questioning included the degree to which patients participated in decision-making, challenges experienced, and suggestions for improving the decision-making process. We analyzed the data using a directed content and thematic approach.

**Results:**

We interviewed 16 patients ranging in age from 28 to 68 years. Nine were male, 10 underwent IPAA, and 6 underwent EI. When it came to participation in decision-making, 11 patients felt independently responsible for decision-making, 3 shared decision-making with the surgeon, and 2 experienced surgeon-led decision-making. Themes regarding challenges during decision-making included lack of support from family, lack of time to discuss options with the surgeon, and the overwhelming complexity of the decision. Themes for ways to improve decision-making included the need for additional information, the desire for peer education, and earlier consultation with a surgeon. Only 3 patients were content with the information used to decide about surgery.

**Conclusions:**

Patients with ulcerative colitis who need surgery largely experience independence when deciding between IPAA and EI, but struggle with inadequate educational information and social support. Patients may benefit from early access to surgeons and peer guidance to enhance independence in decision-making. Preoperative educational materials describing surgical complications and postoperative lifestyle could improve decision-making and facilitate discussions with loved ones.

**Supplementary Information:**

The online version contains supplementary material available at 10.1186/s12876-021-01916-0.

## Background

Up to 30% of patients with ulcerative colitis will require surgery during the course of their illness [1]. Most patients undergo total proctocolectomy followed by either reconstruction of the rectum with ileal pouch-anal anastomosis (IPAA) or permanent end ileostomy (EI) in a 1-, 2- or 3-stage fashion. However, the decision-making process is difficult owing to the complexity of the options, which have very different risk profiles and impact on quality of life. It therefore has been suggested that this decision is well-suited to shared decision-making [2].

Shared decision-making describes a collaborative process between patients and healthcare providers that results in a decision reflecting best medical evidence and the patient’s values and preferences [3]. Shared decision-making has the potential to improve healthcare outcomes by increasing patient knowledge and aligning care with patient values [4]. Further, shared decision-making is considered an important pathway to achieving patient-centered care and improving health care quality [5, 6].

Among inflammatory bowel disease patients, shared decision-making regarding medical management improves health care outcomes, including patient adherence, anxiety, satisfaction, and costs of care [7–9]. Previous studies of medically managed ulcerative colitis patients demonstrate high levels of desire to actively participate in decision making [10]. However, preferences for participating in decision-making are dynamic and for a single patient may change with time or with different decisions [11, 12]. Further, patients may prefer less involvement in decisions they perceive to be higher risk [13]. Therefore, it is not clear whether these same preferences would apply to ulcerative colitis patients who are making surgical decisions.

Following total proctocolectomy, the decision between IPAA and EI is a high stakes decision with permanent implications. In order to support patients in making good surgical decisions, it is important to understand the decision-making process from the patient’s perspective. However, very little is known about the extent to which patients with ulcerative colitis participate in making a decision about surgery or their needs during the decision-making process. We therefore conducted interviews with ulcerative colitis patients who underwent surgery to gain an in-depth understanding of the decision-making process and to identify ways to support patients in deciding between IPAA and EI.

## Methods

### Participants

We enrolled adult patients age 18–70 years with ulcerative colitis who had surgery at the University of California, San Francisco resulting in an IPAA or a permanent EI in any number of stages. Eligible patients completed surgical therapy 4–24 months prior to the interview. We required a minimum of 4 months so that patients had recovered from surgery and would have experience adjusting to living with IPAA or EI. We also limited enrollment to those who completed surgery within the prior 24 months to maximize recall regarding preoperative decision-making processes. The age limit of 70 was chosen to optimize the group where IPAA would be a surgical option [14]. We identified all candidate patients, and then did a selective invitation process (letter followed by telephone call) to balance enrollment of patients into age categories (age 18–30, age 31–50, and age 51–70). In each of these age groups we planned to recruit at least one man, one woman, one patient undergoing permanent end ileostomy and one undergoing IPAA. These categories were developed in an effort to obtain a balanced mix of views and opinions with the hypothesis that patient experiences and perceptions would vary by age, sex, and procedure type.

### Interviews

Semi-structured interviews were conducted by a female member of the team with prior experience conducting qualitative research (RH). The interview guide (Additional file 1) consisted of open- and closed-ended questions refined iteratively by pilot testing with a large team that included an experienced colorectal surgeon (EF), decision scientist (EO), qualitative expert, and gastroenterologist. Interviews took place in person (n = 3) or over the phone (n = 13) and were conducted from a research office. Participants did not have an established relationship with the interviewer, nor were participants given any personal information about the interviewer. At the conclusion of each interview, participants were asked if they would like to discuss anything else about their decision-making experience. Interviews ranged in length from 25 to 58 min. No field notes were taken during or after the interview. Transcripts were not returned to participants for comment or correction. All participants completed survey of basic demographic and disease-specific information, which was recorded in REDCap (Research Electronic Data Capture), a secure web-based application [15]. Participants were offered a small monetary incentive for participating.

### Data analysis

A third party service transcribed the audio recordings verbatim for analysis. Thematic analysis was performed using the method described by Crabtree and Miller [16]. Authors JC, RH, YK, AK, CL, as well as CK (see acknowledgements) independently reviewed transcripts and coded the text to identify themes. We reviewed themes at regular meetings with at least 6 authors in attendance. A final code assignment for each transcript was reached by consensus. We modified the interview guide in response to emerging themes and conducted interviews until thematic saturation was reached as determined by consensus of the authors.

### Ethical considerations

Written informed consent was obtained from all participants. The University of California, San Francisco Committee on Human Research approved all study procedures. This study is reported using the Consolidated Criteria for Reporting Qualitative Research Checklist (COREQ), see Additional file 1.

## Results

We conducted a total of 17 interviews, 16 were included in the analysis. One subject was diagnosed with Crohn’s disease after surgery and was excluded. Participant characteristics are detailed in Table 1. The mean age was 44 years and there were 8 men. Participants completed all stages of surgery a median of 18 months prior to their interview. Ten underwent IPAA and 6 received a permanent end ileostomy (EI). All 6 patients in the permanent end ileostomy group underwent 1-stage surgery. 3 patients in the IPAA group underwent a 2-stage procedure (stage 1: total proctocolectomy with IPAA and diverting loop ileostomy; stage 2: ileostomy reversal). 7 patients in the IPAA group underwent a 3-stage procedure (stage 1: subtotal colectomy with end ileostomy; stage 2: completion proctectomy with IPAA and diverting loop ileostomy; stage 3: ileostomy reversal).

**Table 1:** Participant characteristics

Patient characteristics	Total N	(%)	IPAA (N = 10)	EI (N = 6)
Age				
18–30 years	2	12.5	1	1
31–50 years	8	50.0	6	2
51–70 years	6	37.5	3	3
Sex				
Female	8	50.0	5	3
Male	8	50.0	5	3
Race				
White	15	93.8	10	5
Asian	1	6.2	0	1
Ethnicity				
Hispanic	1	6.2	0	1
Non-Hispanic	15	93.8	10	5
Highest completed education				
High school	6	37.5	2	4
College or more	9	56.3	7	2
Unknown	1	6.2	1	0
Family history of IBD				
Yes	7	43.8	4	3
No	9	56.2	6	3
Subtotal colectomy with end ileostomy prior to definitive surgery				
Yes	7	43.8	7	0
No	9	56.2	3	6
Type of definitive surgery				
TPC with EI	6	37.5	–	–
TPC with IPAA (2 stage)	3	18.8	–	–
CP with IPAA (3 stage)	7	43.8	–	–

*IPAA* Ileal pouch-anal anastomosis, *EI* end ileostomy, *IBD* inflammatory bowel disease, *TPC* total proctocolectomy, *CP* completion proctectomy

### Participation in decision making

When it came to deciding between IPAA and EI, most ulcerative colitis patients expressed active participation in decision-making. Eleven patients reported that they experienced “patient-led” decision-making (where the patient felt independently responsible), 3 shared decision-making with the surgeon, and 2 experienced surgeon-led decision-making (please see Table 2 for representative quotes). Surgeon-led decision-making was used in cases where the surgeon felt the chance of pouch failure was high due to indeterminate colitis (N = 1) or low sphincter tone (N = 1).

**Table 2:** Representative quotes regarding participation in decision making between end ileostomy and ileal pouch-anal anastomosis

Theme	Representative quote
Patient-led	“I think I had it pretty good, in terms of decision time, and options, and the ultimate responsibility to make that decision by myself. I didn't feel as though I was between a rock and a hard place. I could choose either way, and everybody involved would be completely fine with that.”
Shared with surgeon	“I would definitely like to get all the information possible from the surgeon…and I would like to bring my thoughts into it, and have a back-and-forth conversation about it.”
Surgeon-led	“I deferred totally to [the surgeon]. At that time, I hadn't researched enough. I didn't know enough.”

Many patients felt that the decision between IPAA and EI was well-suited to patient-led decision-making:

The surgery is going to change your life. So if you need to make some drastic changes in your life, you might as well be the one in charge of that.

Others expressed that the surgeon’s opinion was an important part of decision making:

I felt like [IPAA] was…the option for me. All the information [the surgeon] gave me, you know, my age, and my health, would increase my chances of success, and again, it would be a better choice for me.

Those who experienced surgeon-led decision making felt comfortable trusting the opinion of the surgeon:

Well, I put all my faith in my doctors. And it's like, you know, they're in the business. I'm not…so I put a lot of faith in them. And when [the surgeon] told me that I'd have problems at my age…well, I just went along with her, because I figured, you know, she's the professional not me.

Seven patients underwent a subtotal colectomy as a first step in treatment. In this group, 6 patients had a passive tone in decision making regarding the choice to have surgery, and in many instances, didn’t see that there was a choice to be made. This sometimes was due to the severity of the illness:

There was really no [choice] - I didn't care. I mean, just do anything, but do it now…just get [the colon] out of me.

The Humira didn’t work. They put me on the TPN [total parenteral nutrition]. And then that’s when they said, ‘We’re going to need the surgery.’ And so it wasn’t like I had a doctor’s appointment, walked in and somebody said, ‘We need to discuss surgery.’

Others expressed needing to trust their physician to save their life:

I had a hemorrhage, so there wasn’t really a decision that I could make…I figured that if I listened to the doctor, or, you know, followed what the doctor’s orders were, that I would be fine.

Five of the 6 patients who experienced passive decision-making regarding subtotal colectomy surgery participated more actively in shared- or patient-led decision making when it came time to deciding between IPAA and end ileostomy. As one patient recalled:

They told me, ‘You either have surgery, or you’re going to get colon cancer.’…For the J-pouch, they all said that was my decision. But in terms of removing part of my colon or all or part of the colon, all of them recommended that it would be much better to have all the colon taken out.

These experiences suggest that in acute illness or in the setting of cancer risk, patients perceived less choice for surgical treatment. In general, patients seemed to be comfortable with this. In contrast, these same patients showed increased engagement in the decision-making process when facing a decision between IPAA and EI. This suggests that patient participation in decision making is dynamic and decision-dependent.

### Challenges to decision making

When discussing challenges that patients encountered during decision-making, three themes emerged. These included patients who felt that they did not have enough time with their surgeon during decision-making, the overwhelming complexity of the decision, and lack of support from family members who did not agree with the patient’s decision (Table 3).

**Table 3:** Representative quotes regarding difficulties during decision-making between end ileostomy and ileal pouch-anal anastomosis

Theme	Representative quote
Not Enough Time with Surgeon	“And which I know that's kind of hard sometimes…[the surgeon] is busy, and it's understandable. So sometimes I felt like there was maybe a little bit shy of conversation.”
Complexity of Decision	“…my ability to process the information was so limited because it was so overwhelming, and there was so much of it.”
Family not Supportive	“My Mom tried to talk me out of [an ileostomy]…she thought my relationship with my husband would change, you know, sexually.”

When it came to lack of support, a number of sub-themes emerged. First, patients had variable experiences with their family members, some having supportive and helpful relationships:

There were no difficulties. My three children thought I should have had it sooner. And you know, there were no problems. Everybody was supportive, especially my husband.

And patients found that specific people were helpful, while others were not:

My Dad's just kind of there, literally, he's just kind of there. My sister, she doesn’t really like to talk about it, especially when, like, she almost fainted when she came in to see me when I was here after my surgery. She doesn't like thinking about me being sick, or knowing anything about it, really. But it was mainly me and my mom. I don't think I've ever really made an executive decision, you know, completely by myself.

It was common that stigma regarding ileostomy surgery was a source of difficulty in feeling supported:

The people that I decided not to include in divulging I was having this kind of surgery were the people who were, like, ‘Oh, that would be the last thing. I don't know what I would do if I had that. That's horrible’…So there have been some people in my life who, again, have this stigma of what this is like, having an ileostomy. And I say that their negativity is what played a role in me having to keep it from them. So as far as my decision-making for the surgery, I decided not to take their opinions in account.

One patient felt comfortable having less involvement from family members:

Well, there was no one to talk it over with. I mean, I had my mom. I mean, no one really understands, either from an intellectual aspect, or a technical aspect. And the emotional part, you know, I never really need any help with that.

Ulcerative colitis patients vary in their desire for social support and in the amount of support available when making a decision between permanent EI and IPAA, highlighting the role for an individual assessment of patient needs during decision making.

### Improving decision making

When discussing ways to improve decision-making, three themes emerged. Themes included a desire to have peer education regarding surgical options, earlier consultation with a surgeon, and the desire for more information prior to making a decision (Table 4).

**Table 4:** Representative quotes regarding ways to improve decision-making between end ileostomy and ileal pouch-anal anastomosis

Theme	Representative quote
Desire peer education	“It would have been a great tool to be able to meet with a group of women and lay out all the questions on paper, you know, and have them generally talk to me about that. It would have been much easier”
Earlier consultation with surgeon	“Earlier discussion of the surgery so that it's not this far off and away foreign ‘it doesn't happen to me’ kind of situation, because I think so many people…had they had a successful surgery when they were healthy, and in a good position to do so, life might be better.”
Desire more information	“I took what they provided me, and I did more in-depth research on my own…they could have given me something with a lot more information in it. And I could have benefited from that.”

Within the theme of desire for more information, 3 subthemes emerged. First, patients expressed a desire to have more information in general terms:

There were quite a few details that I wish I had known before. But I didn't know to ask about them, even.

Others desired more information about complications:

I wanted to hear more details about the complications. And as far as I remember, it wasn't trivialized. But it was kind of, like, not enough attention was paid to discuss that in length.

Still others wished to have more information about what life would be like in the long-term:

I mean, most of the stuff I heard sounded as close to a regular life as you could get. So I don't know. To me, there's lots of realities that I don't feel like are really introduced or just explained.

Only three patients felt content with the information they used to decide about surgery. These patient experiences demonstrate the variable needs of patients as they go through the decision-making process and highlight the need for reliable and relevant information for patients who are making decisions between IPAA and EI.

## Discussion

In this study, we used semi-structured interviews to characterize the process of decision-making between IPAA and EI among surgical patients with ulcerative colitis. We found that patients largely experienced active participation in decision-making between IPAA and EI, but struggled with not having enough time with their surgeon, dealing with unsupportive family members, and feeling overwhelmed by the general complexity of the decision. Patients felt that decision-making could be improved through increased information, access to peer education, and earlier consultation with a surgeon. By focusing on ulcerative colitis patients who have made the difficult decision between IPAA and EI, our study addresses a critical gap in the existing literature, which includes studies on general perceptions of shared decision making [10] and educational needs [17, 18] of non-surgical inflammatory bowel disease patients, or patients who are making a decision between surgery and medical management [19].

Our results demonstrate high levels of patient participation in decision-making about surgery. This finding is not surprising when considering other studies in inflammatory bowel disease patients, which demonstrate high levels of patient interested in shared decision-making about medical management. For example, Baars, et al. found that 98% of inflammatory bowel disease patients reported that it was “very” or “quite” important to participate in decision making regarding medical management of their disease [10]. A study in Japanese inflammatory bowel disease patients showed that 92% of patients felt that shared-decision making was “very” or “quite” important, and that a history of surgery was a predictor of the desire for shared decision-making [20]. Further, our patients were able to achieve high levels of participation in decision-making despite perceived barriers, which also speaks to their motivation to participate. It is important to address these barriers so that patients can participate to their desired level and in a meaningful way. Patient decision aids have promise for supporting shared decision making and three have been developed for inflammatory bowel disease patients [21–23] with further trials underway [24, 25].

Our results further demonstrate that patient participation in decision-making can change with different decisions. In the present study, we found that patients undergoing subtotal colectomy as a first step in their surgical treatment were likely to report a passive role in decision-making regarding the decision to undergo subtotal colectomy, but then later played an active role in making a decision about IPAA vs EI. The reasons for the decreased participation at the time of subtotal colectomy were not purposefully explored in the present study, however, other studies demonstrate that patients making medical decisions tend to defer decision making to physicians when they have more severe disease [20] or disease likely to result in mortality [13]. This finding underscores the importance of reassessing patient desire to participate in decision-making at each step of treatment.

The association between patient education and the desire to engage in shared decision-making has been shown in a variety of disease states [26] including inflammatory bowel disease [8]. The majority of patients in the present study desired increased information prior to making a decision about surgery. The need for improving patient education is particularly relevant in ulcerative colitis patients, because more than 56–62% feel insufficiently informed about their disease [17, 18, 27]. However, patients feel that existing educational materials are not tailored to their needs, including a lack of coverage of important topics, such as long-term recovery, and practical matters, such as returning to exercise, dietary restrictions, and management of stomas [19]. The educational needs of ulcerative colitis patients should be addressed in order to support patients in engaging in decision-making in a meaningful way. Our group previously reported a pilot study demonstrating increased patient knowledge and confidence regarding decision-making among ulcerative colitis patients using a decision aid [22]. It is possible that the identified knowledge gap could be closed through wider dissemination of existing decision aids and web-based sources [28–30], which should be specifically evaluated for information completeness using large groups of ulcerative colitis patients and other stakeholders.

Our study demonstrated that some patients struggled with inadequate support from family members and there was a desire for increased peer support as a mechanism to improve the decision-making process. This support gap has been challenging in inflammatory bowel disease patients because, although other studies show that patients wish to have peer support when they are making decisions [10, 19], structured, regular peer support groups for inflammatory bowel disease often fail [31]. One reason for this finding is that patients find peer support helpful during flares of disease or when they need help with a particular problem as opposed to when they are feeling well [31]. This challenge indicates that a different “on-demand” structure for peer support is needed, such as online groups that patients can utilize when they need specific support, such as around the time of surgery.

The patients enrolled in the present study desired earlier consultation with a surgeon. This finding is in agreement with prior work that shows that more than half of UC patients would have preferred to have an earlier operation [32]. We recommend that surgical consultation not be viewed as a failure of medical management. Involving surgeons early is particularly relevant because a multidisciplinary team-based approach to inflammatory bowel disease is increasingly viewed as important and is recommended by the American College of Gastroenterology and British Society of Gastroenterology [33, 34]. Earlier consultation with a surgeon could also address patient concerns of the overwhelming complexity of the information and not having enough time with the surgeon, as surgical discussions could be spaced out over time and new questions answered as they arise.

This study provides a rich qualitative analysis of decision making between IPAA and EI among patients with ulcerative colitis, however, there are limitations that must be considered when interpreting the results. First, the results are based on a relatively small sample from a single institution. In order to create the generalizability, we performed purposeful enrollment of patients across age groups, sex, and procedure type and ensured saturation prior to completing enrollment. Second, we performed interviews postoperatively after decision-making was completed. Patient postoperative experiences could change the way that patients view their decision-making process, and it may be difficult for patients to recall information from the past. However, we attempted to mitigate these influences by conducting interviews 4–24 months after surgery, a shorter time interval than used in prior studies [19].

## Conclusion

Our findings demonstrate that ulcerative colitis patients play an active role in decision making between IPAA and EI. However, they struggle with obtaining adequate social support and information to support decision-making and perceive these decisions to be overwhelmingly complex. Patients could be better supported in decision-making through a multi-faceted approach that would include increased access to relevant educational resources, surgeons, and peers. Preoperative educational materials should include information about postoperative complications and long-term changes in lifestyle. These materials could help improve decision-making and facilitate discussions with loved ones.

## Supplementary Information

**Additional file 1** Interview Guide.

## Data Availability

The datasets used and/or analyzed during the current study are available from the corresponding author on reasonable request.

## Abbreviations

IPAA: Ileal pouch-anal anastomosis
EI: End ileostomy
IBD: Inflammatory bowel disease
CP: Completion proctectomy
TPC: Total proctocolectomy
TPN: Total Parenteral Nutrition
