# Supplementary material for: Ileostomy or ileal pouch-anal anastomosis for ulcerative colitis: patient participation and decisional needs
Source: BMC Gastroenterol. 2021 Sep 19;21:347. doi: 10.1186/s12876-021-01916-0 (PMC8451075; doi:10.1186/s12876-021-01916-0)
Supplement: Supplementary file 1 — Additional file 1 Interview Guide. [file 12876_2021_1916_MOESM1_ESM.doc]

Study ID:__________________

**INTERVEW GUIDE**

*Note: Text and central questions (in* 12 point*) should be said to / asked of all participants, probe questions* (in 10 point parentheses) *do NOT need to be asked of all participants, and should only be used if needed to elicit participants’ perspectives on the central question.*

*[audio-recorder on]*

***INTRODUCTION:***

“Ok, I’ve turned on the audio-recorder, and we are ready to begin the interview. For my records, I am speaking with study participant ID *xxx* and the date is *xxx*. This interview is being conducted [*by telephone/in-person*].

Thank you again for taking the time to participate in this study. We have discussed the information in the informed consent document. I would like to pause for a moment and make sure that you do not have any additional questions about participating before we start.

As we discussed, the goal of the interview is to learn about your experience with ulcerative colitis, specifically around the time you were making decisions about surgery. By speaking with you, we hope to learn how we can best support others who need to make similar decisions.

If you feel uncomfortable at any time during the interview, please let me know. You do not have to answer any questions that make you feel uncomfortable and we can take a break or end the interview at any time.

Do you have any questions before we begin?”

***SECTION 1: Experience with ulcerative colitis***

*Goals: To explore the participant’s experience being diagnosed and living with ulcerative colitis. To understand the events leading up to the decision to have surgery.*

-What was it like to live with ulcerative colitis?

1. (What was it like when you were first diagnosed?)
2. (How have things changed over time?)
3. (How do you think your life has changed as a result of ulcerative colitis?)

-Tell me what it was like when you first realized you might need surgery.

1. (How did it feel?)
2. (Was there anything that worried you about surgery?)
3. (Why did you decide to have surgery?)

***SECTION 2: Experience making decisions about surgery***

*Goals: To understand the process used to come to a decision about which type of surgery to have.*

-Walk me through the process of figuring out what type of surgery you were going to have.

1. (How much input did you have?)
2. (Did you wish to have more or less responsibility for the decision than you did?)
3. (What role did your doctors or others play in the decision?)
4. (What were the most important factors in your decision? Did you discuss these with your doctor?)
5. (Was there anything that would have helped you feel more confident about the decision?)
6. (Who did you get advice from?)
7. (What resources, if any, did you use to get information about surgery? How helpful was each?)
8. (Would you change anything about the decision making process if you could go back in time?)

***SECTION 3: Lessons learned***

*Goals: To understand what life is like after surgery and how we can better prepare patients before surgery.*

-What are things like now that you have had surgery?

1. (In what ways has your life changed?)
2. (In what ways have your relationships changed?)
3. (Have there been any surprises about life after surgery?)
4. (What advice would you give other people before surgery knowing what you know now?)
5. (If you could go back in time, would you do anything differently?)
6. (What do you think are the most important things people should know before they have surgery?)

-Do you feel satisfied with the surgery?

1. (Why/Why not?)

***SECTION 4: Closing***

*Goals: To obtain participant’s views on other aspects of surgical decision making that were not addressed.*

-Are there any other aspects of your experience having surgery that you would like to share?

-Are there any final thoughts you would like to share with me before we end the interview?

“Thank you again for participating in this study and sharing your insight with me. As we discussed, the goal of this research is to improve the way people with ulcerative colitis prepare to make decisions about surgery. Your thoughts are essential to helping us accomplish this goal.”

IF PHONE INTERVIEW: “Please take a few minutes now to seal the background information survey and signed informed consent document in the envelope and put it in the mail to us today.”

*[Leave audio-recorder on until you and participant are leaving the interview room or until the phone is hung up.]*
